# Constitutive activation of STAT3 and STAT5 is present in the majority of nasopharyngeal carcinoma and correlates with better prognosis

**DOI:** 10.1038/sj.bjc.6601003

**Published:** 2003-07-15

**Authors:** J-R Hsiao, Y-T Jin, S-T Tsai, A-L Shiau, C-L Wu, W-C Su

**Affiliations:** 1 Institute of Clinical Medicine, College of Medicine, National Cheng Kung University, 1 Doshiue Road, Tainan 701, Taiwan; 2 Department of Otolaryngology, National Cheng Kung University Hospital, 138 Sheng-Li Road, Tainan 704, Taiwan; 3 Department of Pathology, National Cheng Kung University Hospital, 138 Sheng-Li Road, Tainan 704, Taiwan; 4 Department of Microbiology and Immunology, College of Medicine, National Cheng Kung University, 1 Dashiue Road, Tainan 701, Taiwan; 5 Department of Biochemistry, College of Medicine, National Cheng Kung University, 1 Dashiue Road, Tainan 701, Taiwan; 6 Department of Internal Medicine, National Cheng Kung University Hospital, 138 Sheng-Li Road, Tainan 704, Taiwan

**Keywords:** signal transducers and activators of transcription (STAT), nasopharyngeal carcinoma (NPC), Epstein–Barr virus (EBV)

## Abstract

Constitutively activated signal transducers and activators of transcription (STAT) factors, in particular STAT1, STAT3 and STAT5, have been demonstrated in a variety of human tumours and cancer cell lines. However, data on the expression of these STATs in nasopharyngeal carcinoma (NPC) are limited. In this study, the expression patterns of STAT1, STAT3 and STAT5 were immunohistochemically examined on the archival specimens from 61 patients with NPC. Staining results of each STATs were then correlated with the clinical parameters and prognosis of these patients. The results showed that constitutive activation of STAT3 and STAT5 was detected in the majority, 70.5 and 62.3%, respectively, of the 61 tumour specimens. Furthermore, coexpression of activated STAT3 and STAT5 was found in 54.1% of the specimens. In contrast, constitutive activated STAT1 could only be detected in 8 (13.1%) cases. Surprisingly, following radiotherapy, patients with constitutive STAT5 activation, or activation of both STAT3 and STAT5, had better disease-free survival and overall survival than those without activated STAT5. To our knowledge, this is the first report providing the overall expression patterns and prognostic significance of specific STATs in NPC.

Signal transducers and activators of transcription (STAT) proteins comprise a family of transcription factors and function as downstream effectors of many cytokine and growth factor receptors (Darnell, 1997). Signalling pathways mediated by STATs are critical for many normal cellular functions, including embryonic development, organogenesis, immunological interaction, growth, differentiation and survival (Buettner *et al*, 2002). During cytokine or growth factor stimulation, STATs are tyrosine phosphorylated, dimerise, and translocate into the nucleus to regulate target gene expression (Darnell, 1997). The activation duration of STATs under physiological conditions is usually temporary and is tightly regulated by a number of cellular mechanisms, such as tyrosine dephosphorylation, ubiquitin/proteosome-mediated degradation, negative feedback loop mediated by CIS/SOCS/JAB/SSI family of proteins, or inhibition of STAT DNA-binding activity through association with protein inhibitor of activated STAT (PIAS) proteins (Lin and Leonard, 2000). However, constitutively activated STATs, especially STAT1, STAT3 and STAT5, have been found in a variety of human tumours and cancer cell lines, including blood malignancies and solid tumours (Turkson and Jove, 2000). In various tumour cell lines, persistent signalling of specific STATs, in particular STAT3 and STAT5, has been shown to stimulate cell proliferation and prevent apoptosis through upregulating a number of target genes, such as c-Myc, cyclins and bcl-x. In contrast, inhibition of constitutively activated STAT3 or STAT5 leads to growth suppression or apoptosis (Buettner *et al*, 2002).

Nasopharyngeal carcinoma (NPC) is endemic in South-east Asia and closely associated with Epstein–Barr virus (EBV) (Tsai *et al*, 1996). Epstein–Bars virus resides in NPC cells in a status called type II latency, with only a few latent genes expressed (Brooks *et al*, 1992). One of these latent genes, latent membrane protein 1 (LMP-1), has been shown to have transforming potential (Baichwal and Sugden, 1988; Kaye *et al*, 1993) and is implicated in the oncogenesis of various EBV-associated malignancies, including NPC. LMP-1 can also interact with Janus Kinase 3 (JAK3) and activate STAT proteins (Gires *et al*, 1999). In a recent study, the JAK/STAT pathway was shown to play a role in maintaining *in vivo* latency of EBV (Chen *et al*, 1999). Aberrant STAT activation has also been proposed as a necessary and predisposing event for EBV-driven tumorigenesis in immunocompetent individuals (Chen *et al*, 2001).

Although the presence of constitutive STAT1, STAT3 and STAT5 activation has been demonstrated in NPC tissue (Chen *et al*, 2001), data on the levels of expression of these STATs in NPC are lacking. Thus, whether expression of these constitutively activated STATs affects prognosis after treatment remains unclear. This study used immunohistochemical methods to examine the expression pattern of STAT1, STAT3 and STAT5 in biopsy specimens of patients with NPC and correlated the results with clinical parameters of these patients.

## MATERIALS AND METHODS

### Patients and specimens

A total of 61 NPC patients with complete follow-up records and available archival nasopharyngeal biopsy specimens were recruited in this study. All of the 61 patients, including 49 males and 12 females, were histologically confirmed to have WHO type II (nonkeratinising) or WHO type III (undifferentiated) NPC (Shanmugaratnam *et al*, 1979). The clinical status of these patients was determined using the 1997 UICC/AJCC staging system (Sobin and Wittekind, 1997) after reviewing their clinical records and image studies which included chest X-ray, abdominal sonography, bone scan and CT scan. All patients were previously untreated and received radiation treatment with curative intention between 1990 and 2000 at the Department of Radiation Oncology of National Cheng Kung University Hospital. In all, 56 patients received radiotherapy as their sole treatment modality. The other five patients received concurrent chemotherapy with bolus infusion of cisplatin 100 mg m^−2^ day^−1^ on the beginning day of weeks 1, 3 and 5 after the start of the radiotherapy regimen. All 61 patients completed the full-course of radiation therapy within 9 weeks without any interruption and received regular follow-up at our hospital. Among the 61 patients, 41 remained disease-free for a median follow-up period of 57.1 months, ranging from 26.3 to 151 months. In total, 20 patients suffered from loco-regional relapse and/or distant metastasis after treatment, including nine patients with local recurrence (one also with distant metastasis), eight patients with recurrence in the neck (five also with distant metastasis), two patients with both local and neck recurrence (one also with distant metastasis) and one patient with distant metastasis without loco-regional relapse. All the archival nasopharyngeal biopsy specimens used in this study were obtained from the Department of Pathology of National Cheng Kung University Hospital.

### Immunohistochemical study

Serial 5-*μ*m histological sections were cut, mounted on glass slides coated with 3-aminopropyltriethoxysilane, and air-dried overnight at room temperature. The sections were then deparaffinised in xylene and rehydrated in ethanol. Haematoxylin and eosin (H&E) staining was first performed in each specimen to confirm the presence of tumour cells. Endogenous peroxidase activity was then blocked with methanol containing 3% H_2_O_2_ for 15 min. For all sections used in this study, the antigen retrieval procedure was performed by immersing the slides in citrate buffer (pH 6.0) and then heating the slides in a microwave oven for 10 min. The sections were then incubated with primary antibodies using 1 : 25 dilution for STAT1, STAT3 and STAT5 (mouse anti-human IgG1, Santa Cruz, CA, USA) at 4°C overnight, followed by staining with Universal Immuno-peroxidase polymer (UIP) solution (Simple Stain MAX PO MULTI, Nichirei, Tokyo, Japan) for 30 min. The sections were finally reacted with AEC (3-amino-9-ethylcarbazole) substrate solution (DAKO, Glostrup, Denmark) and then counterstained with haematoxylin before being mounted. Non-human reactive mouse IgG1 (DAKO, Glostrup, Denmark) was used as an isotype negative control.

The sections were then observed under a light microscope by a pathologist (IT Jin) who was blinded to the clinical characteristics of the patients. The degree of expression of each STAT was then determined independently with the following rules. Intranuclear staining of tumour cells was considered to indicate the presence of constitutively activated STATs. Using high magnification power (× 400), five representative fields in each section were evaluated. In total, 100 tumour cells were counted in each field. In sections containing less than five tumour nests, all tumour nests were evaluated. The percentage of immunoreactive tumour cells with nuclear staining was then calculated. When equal or more than 20% of the counted tumour cells on one slide showed identifiable nuclear staining, the slide was classified as ‘positive’ for significant constitutive STATs activation. If less than 20% of tumour cells showed a nuclear staining pattern, the slide was considered to show no significant constitutive activation of STATs and was classified as ‘negative’. Staining results of the specimens were then correlated with the clinical characteristics of the patients.

### Statistical analysis

*χ*^2^ test was used to calculate the significance of the relation between expression of each STAT and clinical characteristics. The Kaplan–Meier method was used in the survival analysis. Log-rank test was used to calculate the significance of differences in the survival analysis. Cox's proportional hazards regression model was used to study the influence of covariates on survival time. A probability level of less than 0.05 (*P*<0.05) was considered to indicate a significant difference.

## RESULTS

### Constitutive activation of STAT3 and STAT5 in the majority of NPC specimens

Both STAT3 and STAT5 were significantly activated in a high proportion of the NPC specimens included in this study. Significant constitutive STAT3 activation was noted in 43 (70.5%) of the 61 samples studied, while significant constitutive STAT5 activation was noted in 38 (62.3%) of the 61 specimens (Table 1

**Table 1:** Patient characteristics and the staining results of STAT3 and STAT5

		**Constitutive STAT3 activation**			**Constitutive STAT5 activation**		
		**(+)**	**(−)**	***P*-value**	**(+)**	**(−)**	***P*-value**
Number of specimens		43 (70.5%)	18 (29.5%)		38 (62.3%)	23 (37.7%)	
*Clinical parameters*							
Age	Mean±s.d. (years)	49.3±11.9	44.6±11.0	0.15	47.5±11.4	48.6±12.4	0.71
Sex	Male	34	15	0.98	29	20	0.49
	Female	9	3		9	3	
T	T1	13	6	0.31^a^	13	6	0.55^a^
	T2	25	12		23	14	
	T3	0	0		0	0	
	T4	5	0		2	3	
N	N0	17	7	0.73^b^	15	9	0.84^b^
	N1	18	9		16	11	
	N2	6	1		6	1	
	N3	2	1		1	2	
Stage	I	7	4	0.46^c^	8	3	0.98^c^
	II	26	12		22	16	
	III	5	1		6	0	
	IV	5	1		2	4	
WHO subtype	II	14	7	0.37	11	10	0.37
	III	29	11		27	13	
Treatment modality	R/T only	39	17	0.85	34	22	0.71
	Concurrent C/T+R/T	4	1		4	1	
							
*Prognosis after treatment*							
Disease-free		32	9	0.12	30	11	0.026*
Recurrence of disease		11	9		8	12	

a T1+T2 *vs* T3+T4.

b N0+N1 *vs* N2+N3.

c Stage I+II *vs* Stage III+IV.

**Figure 1:**
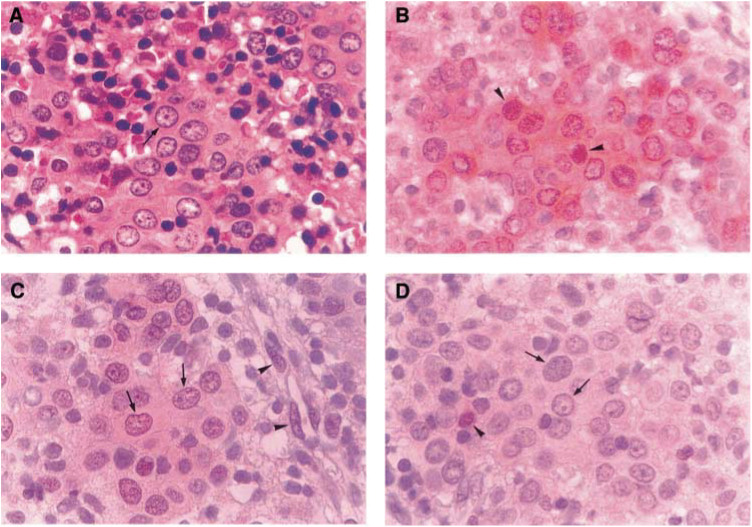
Staining of STAT1, STAT3 and STAT5 on serial pathological sections of a NPC biopsy specimen. (**A**) H&E stain. Large, vesicular nucleus with prominent nucleolus (arrow) is noted in majority of the tumour cells. (**B**) Strong intranuclear (arrowheads) and cytoplasmic staining of STAT3. (**C**) intranuclear (arrows) and faint cytoplasmic staining of STAT5. Nuclear STAT5 staining is also noted in endothelial cells of a nearby vessel (arrowheads). (**D**) No nuclear staining of STAT1 in the tumour cells (arrows). Nuclear staining of STAT1 is noted in an infiltrating lymphocyte (arrowhead) (original magnification × 1000).

* Statistically significant (*P*<0.05).

). In contrast, constitutive STAT1 activation could only be detected in eight (13.1%) of the 61 cases. The intensity of intranuclear staining varied among the specimens, and differences of staining intensity were also noted among tumour nuclei within the same histological section. However, strong intranuclear staining, or intranuclear and cytoplasmic staining, was noted in 31 of the 43 specimens classified as ‘positive’ for significant constitutive STAT3 activation (Figure 1A, B

) and in 32 of the 38 specimens classified as ‘positive’ for significant constitutive STAT5 activation (Figure 1C). In contrast, most of the specimens showed no intranuclear staining of STAT1 protein in tumour cells (Figure 1D).

### Frequent coactivation of both STAT3 and STAT5

Among the 43 specimens classified as ‘positive’ for significant STAT3 activation, 33 were also classified as ‘positive’ for STAT5 activation. Among the remaining 18 specimens that were classified as ‘negative’ for STAT3 activation, 13 were also classified as ‘negative’ for constitutive STAT5 activation (Table 2

**Table 2:** Results of STAT3 and STAT5 staining on the 61 NPC specimens

		**Constitutive STAT3 activation**		
		**(+)**	**(−)**	**Total**
Constitutive STAT5 activation	(+)	33 (54.1%)	5 (8.2%)	38
	(−)	10 (16.4%)	13 (21.3%)	23
				
Total		43	18	61

). The proportion of specimens with same STAT3 and STAT5 classification was 75.4% (46 out of 61), suggesting that STAT3 and STAT5 might be coactivated.

### Constitutive activation of STAT5, or both STAT3 and STAT5, correlates with better prognosis

**Figure 2:**
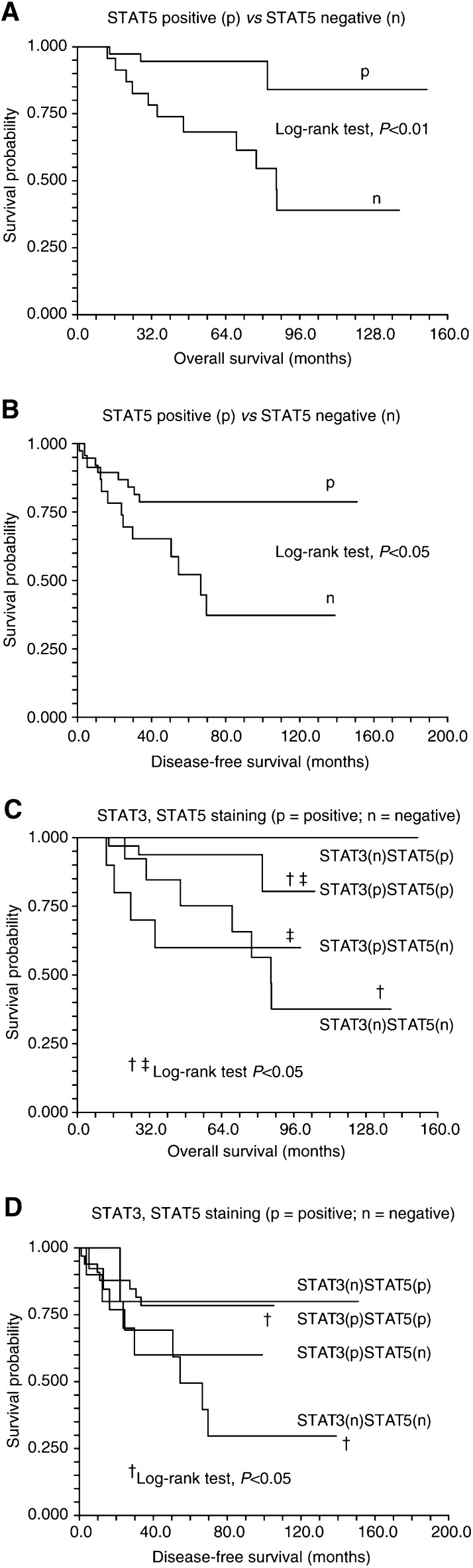
Survival of NPC patients as a function of the staining results of STAT5, or both STAT3 and STAT5: (**A**) Overall survival as a function of results of STAT5 staining, (**B**) disease-free survival as a function of results of STAT5 staining, (**C**) overall survival as a function of results of both STAT3 and STAT5 staining, and (**D**) disease-free survival as a function of results of both STAT3 and STAT5 staining.

Among the 61specimens stained for STAT5 protein, 38 specimens were classified as ‘positive’ and 23 as ‘negative’ for significant constitutive STAT5 activation. As shown in Table 1, no difference was found in the clinical parameters of these two groups of patients, including age, sex, TNM stage, WHO subtype classification and treatment modalities. However, comparison of treatment outcome among these two groups revealed that patients whose biopsy specimens showed significant constitutive STAT5 activation had better prognosis after treatment (*χ*^2^ test, *P*=0.026). Patients who had no significant constitutive STAT5 activation on histological sections were more likely to suffer from loco-regional relapse and/or distant metastasis. On the other hand, the STAT1 and STAT3 staining results showed no such correlation (*χ*^2^ test, *P*=0.36 and 0.12, respectively). This result still held true in the survival analysis. Patients whose biopsy specimens showed significant constitutively activated STAT5 also had better disease-free survival and overall survival than those patients who had ‘negative’ results on their biopsy specimens (Figure 2A, B

; log-rank test, *P*<0.01 and <0.05). Since activation of both STAT3 and STAT5 was frequently noted in the same specimen, we then explored whether this coactivation could have an impact on treatment outcome. Interestingly, patients who had ‘positive’ staining results for both activated STAT3 and STAT5 (33 patients) had significantly better overall survival and disease-free survival than those are ‘negative’ for both staining (13 patients) (Figure 2C, D; ^†^log-rank test, *P*<0.05). However, the double-positive group did not significantly predict better prognosis than the STAT5 positivity only group, both in overall survival and disease-free survival. On the contrary, the double-positive group significantly predicted better prognosis than the STAT3 positivity only group in overall survival (Figure 2C, ^‡^log-rank test, *P*<0.05), implying that STAT5 is more important in predicting prognosis than STAT3. In figure 2C and D, although the STAT5 positivity only group has a favourable survival curve than the other groups, there was no statistical significance detected. This might be due to the small number of patients in the STAT5 positivity only group (five patients). To clarify whether the better prognostic prediction was due to STAT5 positivity only or due to simultaneous activation of STAT3 and STAT5, multivariate analysis was performed. In the Cox's regression model, only STAT5 was selected to be an independent variable for both overall survival (hazard ratio 0.17, *P*=0.008) and disease-free survival (hazard ratio 0.37, *P*=0.03), suggesting that STAT5 is the major determinant of better prognosis.

## DISCUSSION

Constitutively activated STATs have been detected in a variety of human tumours and cancer cell lines, including blood malignancies and solid tumours. Most studies demonstrating constitutive activation of STATs in solid human malignancies have used either Western blot analysis and/or electrophoretic mobility shift assay (EMSA) as the main methodology. However, considering the ‘lymphoepithelioma-like’ nature of NPC (Shanmugaratnam *et al*, 1979), applicability of these methods might be confounded by the infiltrating lymphocytes in this tumour and is not suitable under such conditions. Therefore, we decided to use immunohistochemical methods to study the expression patterns of STATs in NPC. The suitability of this strategy is further supported by the fact that, in addition to their presence in lymphocytes (Figure 1D), constitutively activated STATs are also noted in other nontumour cells (Figure 1C).

A recent study found persistent STAT1, STAT3 and STAT5 activation in NPC tissue (Chen *et al*, 2001). However, insufficient data have been reported to provide an overall picture of the expression of these STATs in NPC. This study has provided the first evidence that significant constitutively activated STAT3 and STAT5 are present in over half of NPC patients (Table 1), while STAT1 activation is present in only a minor proportion of these patients. The intranuclear staining intensity of STAT3 and STAT5 was strong in many of the tissue specimens (Figure 1), suggesting that STAT signalling is very active in this malignancy. We also noted that constitutively activated STAT3 and STAT5 frequently coexisted in the same specimen. It has been shown that different STATs can be activated by the same ligand and/or intracellular tyrosine kinase (Ruff-Jamison *et al*, 1994; Carlesso *et al*, 1996; Olayioye *et al*, 1999). Simultaneously persistent STATs activation is also noted in a variety of human cancers (Turkson and Jove, 2000). As many cellular genes regulating cell cycle and apoptosis, such as c-Myc, cyclin D and bcl-x are downstream targets of STAT3 and STAT5 (Buettner *et al*, 2002), it is reasonable to speculate that persistent signalling of STATs, particularly STAT3 and STAT5, may play a role in tumorigenesis of NPC.

Although STAT1 activation has been reported in some tumours and cell lines, STAT1 activation is associated with tumour suppression rather than proliferation in most conditions (Buettner *et al*, 2002). In a recent study, activated STAT1 was also shown to negatively regulate angiogenesis, tumorigenicity and metastasis of tumour cells (Huang *et al*, 2002). STAT1 deficiency has also been found in a variety of tumour cell lines and this deficiency is responsible for the lack of INF-γ-mediated tumour suppression effects (Wong *et al*, 1997; Abril *et al*, 1998; Sun *et al*, 1998; Pansky *et al*, 2000). Therefore, it was not unexpected that constitutively activated STAT1 was found in only eight (13.1%) of the 61 NPC specimens. In our study, only one of the 20 patients suffering from disease recurrence had significant STAT1 activation.

The presence of constitutively activated STAT5 and coactivation of STAT3 and STAT5 was correlated with better prognosis in this study. Using Cox's regression model, STAT5 was identified to be an independent prognosis-predicting factor. Although STAT3 was found in 70.5% of these NPC patients, it is not an independent prognostic factor for survival. The lack of statistical significance for STAT3 might result from the frequent co-activation of STAT3 and STAT5, or the relative small number of patients in this series. It is also possible that STAT5 is truly the only determinant of better prognosis. The prognostic significance of each STATs in NPC deserves further investigation.

In most conditions, activation of STAT3 or STAT5 up-regulates cell cycle progression and antiapoptotic genes in cells. Therefore, recognizing the constitutive activation of STAT5 as a good prognostic factor is out of our expectation. However, in a number of different studies, activated STATs have been reported to play a role in differentiation and apoptosis (Bromberg and Darnell, 2000). Activated STAT3 has been proposed to facilitate mammary gland involution by inducing extensive epithelial apoptosis through upregulating IGFBP-5 (Chapman *et al*, 1999). Transfection of a constitutively activated STAT5 mutant into an IL-3-dependent Ba/F3 cell line induces expression of bcl-xL and pim-1 and renders the cell line factor-independent. However, IL-3 treatment of the factor-independent cell line resulted in apoptosis within 24 h, or differentiation followed by cell death. This apoptosis might have been due to the concomitant upregulation of JAB/SOCS-1/SSI-1 and p21 by the super-active STAT5 signaling (Nosaka *et al*, 1999). Thus, differences in the fate of cells might be determined by the activation intensity and duration of specific STATs and may be cell-type specific. It has been shown that cellular STATs can also be activated through interactions between JAK3 and LMP-1 protein of EBV (Gires *et al*, 1999). LMP-1 can also upregulate expression of epidermal growth factor receptor (EGFR) (Miller *et al*, 1995), which may then modulate expression of specific STATs upon ligand stimulation (Petersen and Haldosen, 1998; Kloth *et al*, 2002; Leong *et al*, 2002). Interestingly, in line with our findings, despite the fact that LMP-1 of EBV is known for its transforming ability and promotes cellular proliferation in various studies (Baichwal and Sugden, 1988; Kaye *et al*, 1993), tumours with LMP-1 expression tend to have better prognosis in EBV-associated malignancies such as Hodgkin's disease (Montalban *et al*, 2000; Glavina-Durdov *et al*, 2001) and NPC (Hu *et al*, 1995). Regarding the intimate association between STATs and NPC, further clarification of the relation between STAT signalling and the gene expression pattern of EBV may shed light on the pathogenesis of this unique malignancy and might have future therapeutic applications.

In conclusion, by using immunohistochemical methods, we demonstrated that constitutive activation of STAT3 and STAT5 was present in the majority of biopsy specimens from patients with NPC. Besides, constitutive coactivation of STAT3 and STAT5 was frequently noted in the same specimen. By Cox's regression analysis, activation of STAT5 is an independent factor that predicts better prognosis in NPC patients after radiotherapy.
